# Facile Synthesis of Functional Mesoporous Organosilica Nanospheres and Adsorption Properties Towards Pb(II) Ions

**DOI:** 10.3390/nano15020136

**Published:** 2025-01-17

**Authors:** Liping Deng, Shichun Gu, Ruyi Wang, Yapeng He, Hairong Dong, Xue Wang

**Affiliations:** 1 Yunnan Key Laboratory of Modern Separation Analysis and Substance Transformation, College of Chemistry and Chemical Engineering, Yunnan Normal University, Kunming 650500, China; 2 Faculty of Metallurgical and Energy Engineering, Kunming University of Science and Technology, Kunming 650093, China

**Keywords:** one-step synthesis, mesoporous organosilica nanospheres, sulfhydryl modification, dual template, adsorption of Pb^2+^

## Abstract

We successfully synthesize monodisperse sulfhydryl-modified mesoporous organosilica nanospheres (MONs-SH) via one-step hydrolytic condensation, where cetyltrimethylammonium chloride and dodecyl sulfobetaine are employed as dual-template agents with (3-mercaptopropyl)triethoxysilane and 1,2-bis(triethoxysilyl)ethane as the precursors and concentrated ammonia as the alkaline catalyst. The prepared MONs-SHs deliver a large specific surface area (729.15 m^2^ g^−1^), excellent monodispersity, and homogeneous particle size. The introduction of ethanol into the reaction systems could expand the particle size of the synthesized MONs-SH materials from 18 to 182 nm. Moreover, the successful modification of -SH groups endowed MONs-SHs with an excellent adsorption capacity (297.12 mg g^−1^) for Pb^2+^ ions in aqueous solution through ion exchange and complexation function. In addition, the established isotherm model and kinetic analyses reveal that the adsorption of Pb^2+^ ions on MONs-SHs follows the secondary reaction kinetic models, where both physisorption and chemisorption contribute to the adsorption of Pb^2+^ ions. The favorable recyclability of MONs-SHs is demonstrated with the maintained adsorption efficiency of 85.35% after six cycles. The results suggest that the synthesized MONs-SHs exhibit considerable application prospects for effectively eliminating Pb^2+^ ions from aqueous solutions.

## 1. Introduction

Due to the continuous development of mining, metal smelting, metal plating, and battery manufacturing industries, heavy metal pollution has emerged as one of the most serious pollution problems in water treatment [1,2]. Heavy metals in industrial wastewater accumulate in animal and plant bodies through food chain activities, even at low concentrations, and eventually pose a potential risk to the health of humans [3,4]. This is due to their toxicity, persistence, and non-biodegradability. Specifically, as one of the heavy metals widely employed in industrial production, lead can cause damage to human kidneys, nervous system, digestive system, etc., and increase the risk of mutagenicity and carcinogenicity in aquatic organisms and human beings [5,6]. Therefore, the selection of suitable treatment approaches for the elimination of Pb^2+^ from aquatic solutions is essential. Currently, the common methods for eliminating Pb^2+^ ions from water-based solutions mainly include chemical precipitation, solvent extraction, adsorption, membrane separation, ion exchange, etc. [7,8,9]. Among the above approaches, the adsorption method has been commonly utilized in the wastewater treatment industry due to its simple operation, low cost, environmental friendliness, high efficiency, high reliability, and generalizability. Hence, designing and synthesizing low-cost and environmentally friendly methods with excellent adsorption efficiency for degreasing Pb^2+^ ions is urgent for water purification.

So far, a series of functional materials, including metal–organic framework (MOF) materials, activated carbon, biochar, graphene oxide, and mesoporous silicon nanomaterials, have been employed as adsorbents to adsorb lead ions [10,11,12]. For example, Xu et al. successfully prepared phosphoric acid-modified dendritic biochar (PTBB) to improve the elimination efficiency of biochar for Cd^2+^ and Pb^2+^ ions from aqueous solution [13]. The results suggested that the highest adsorption capacity of Cd^2+^ and Pb^2+^ on PTBB reached 98.25 mg g^−1^ and 127.5 mg g^−1^, respectively, which was 1.5 and 1.3 times greater than that of initial biochar. In addition, Ruan et al. successfully prepared Zr-based MOF using 4,6-diamino-2-mercaptopyrimidine, and the synthesized material was utilized for the adsorption of Pb^2+^ from aqueous solution. The results showed that the maximum adsorption was up to 296.5 mg g^−1^ at an optimum pH of six [14]. Among many adsorbent materials, silicon-based mesoporous materials have garnered significant interest due to their features of easily modifiable surf ace groups, substantial specific surface area, and adjustable pore size. The outstanding adsorption performance of silicon-based mesoporous materials has been well demonstrated [15,16]. As a special class of silicon-based mesoporous materials, mesoporous organosilica nanomaterials possess the dual advantages of silicon-based mesoporous materials and organic skeletons. Due to the homogeneous distribution of organic groups throughout the entire framework, numerous adsorption-related applications and exceptional adsorption capacity can be achieved [17,18,19]. Furthermore, several substrates decorated with sulfhydryl groups have been tested for the adsorption of heavy metal cations in previous studies, demonstrating that the sulfhydryl groups have a high affinity for many cations [20]. In addition, the sulfhydryl-modified nanomaterials show excellent performance in the adsorption capacity and recycling of heavy metal ions. This is due to their excellent complexation ability toward toxic ions such as Pb^2+^ and Cd^2+^ [21]. Thus, the sulfhydryl-modified mesoporous organosilica nanomaterials have attracted increasing attention for their promising applications in treating water contaminants.

In this paper, sulfhydryl-functionalized mesoporous organosilica nanospheres (MONs-SH) are successfully prepared by a one-step double-template method, using cetyltrimethylammonium chloride (CTAC) and dodecyl sulfobetaine (SB-12) as double-templating agents, ammonia as the alkaline catalyst, and (3-mercaptopropyl)triethoxysilane and 1,2-bis(triethoxysilyl)ethane as organosilicon sources. The adsorption performance towards Pb^2+^ ions on a MONs-SH adsorbent is probed. During the experiments, we examined the influence of the introduction of ethanol into water systems on the particle size of MONs-SHs. The adsorption behavior of Pb^2+^ ions on MONs-SHs is explored by investigating the initial concentration of Pb^2+^, the reaction time, the solution pH, and the reaction temperature. The adsorption mechanism is further evaluated by fitting different adsorption isotherm models and kinetic processes.

## 2. Materials and Methods

### 2.1. Preparation of the Samples

For MONs-SHs, 0.32 g of CTAC and 0.335 g of SB-12 were dispersed in the mixture solution containing 20 mL ethanol and 80 mL H_2_O, and ultrasonically dispersed for 5 min at room temperature in an ultrasound bath (XM-P102H, Xiaomei Ultrasound Instrument, Kunshan Co., Ltd., Kunshan, China) with 40 kHz frequency and 300 W power to obtain a colorless and transparent solution, which was placed in a 45 °C water bath and stirred for 15 min. Then, 1.6 mL of NH_3_·H_2_O (25–28 wt%), 0.4 mL of MPTES, and 0.8 mL of BTEE were sequentially added with continuous stirring, and continued to react for 4 h. The obtained mixture was centrifuged and washed with deionized water and ethanol three times. Then, the obtained sample was extracted using acidic ethanol to remove the template agent, where the sample was added to 100 mL ethanol and 0.25 mL HCl (37%) and refluxed at 80 °C for 2 h. The above steps were repeated three times. Finally, the templating agent-removed samples were dried in an oven at 60 °C.

For MONs-SHs synthesized in an aqueous system, only 20 mL of ethanol was replaced by an equal volume of deionized water, and other steps were the same as the above procedure.

During the preparation of MONs, MPTES was not added during the synthesis, and the rest of the steps was the same as those for the preparation of MONs-SHs.

### 2.2. Adsorption Performance Tests

The adsorption capacity was determined by dispersing 10 mg of the adsorbent in 10 mL different concentrations of Pb^2+^ solutions at pH 7.0, where the whole reaction time was 60 min. Finally, the Pb^2+^ adsorption capacity was determined using the EDTA titration method. In addition, 0.02 mol L^−1^ of Pb^2+^ solution was selected to further investigate the effects of temperature, time, and pH solution on the adsorption performance of the adsorbents.

#### Titration of Pb^2+^ with EDTA

The concentration of Pb^2+^ was estimated using the EDTA titration method. A total of 1 mL of lead solution (0.00 mol L^−1^–0.04 mol L^−1^) and 10 mL of deionized water were mixed in an erlenmeyer flask. The pH value of the solution was adjusted to 5.0–6.0 by using hexamethylene tetramine and acetic acid. Then, one drop of xylenol orange solution (5 mg mL^−1^) was added. After being titrated with EDTA, the color of the solution changed from purplish red to bright yellow (Figure S1) [22].

## 3. Results and Discussions

### 3.1. Morphological and Structural Characterizations

**Figure 1:**
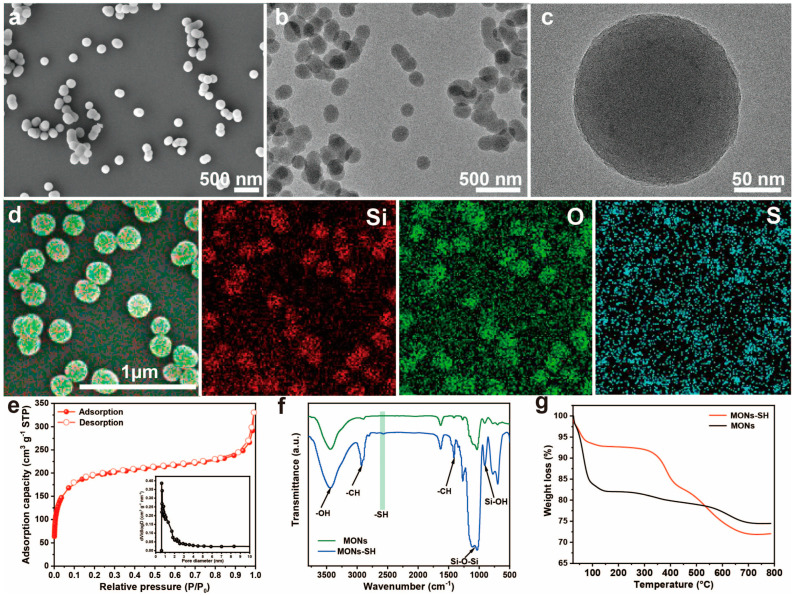
(**a**) SEM image, (**b**,**c**) TEM images, (**d**) element mappings, (**e**) N_2_ adsorption isotherm and pore-size distribution curves of MONs-SHs prepared in an ethanol–water system, (**f**) FTIR spectra, and (**g**) TG curves of MONs-SHs and MONs.

Figure 1a displays the SEM image of the synthesized MONs-SHs in an ethanol–water system. As observed, the nanoparticles exhibit the features of spherical morphology, homogeneous particle size, and good monodispersity. The diameter of the nanoparticles was estimated to be about 182 nm (Figure S2). Figure 1b,c presents the TEM images of MONs-SHs, which clearly reflect the pore structure of the particles. In addition, when BTEE is not added, the white colloidal solution could not be obtained, as shown in Figure S3. From the elemental mapping of the MONs-SH sample in Figure 1d, it can be seen that the chemical composition of MONs-SHs consists of Si, O, and S elements, which are evenly distributed in nanoparticles. In addition, based on the EDS spectra, the weight content of Si, O, and S elements is 54.8 wt%, 33.4 wt%, and 11.7 wt%, respectively, without considering C and H elements, suggesting that Si and O are the main elements (Figure S4). The N_2_ adsorption–desorption isotherms of MONs-SHs suggest the characteristics of the type II curve based on the classification of physisorption isotherms by the International Union of Pure and Applied Chemistry, and adsorption behavior occurs under high relative pressures P/P_0_ of about 0.9, revealing the micro-mesoporous features of the sample [23], as shown in Figure 1e. The BET surface area of the sample was 729.15 m^2^ g^−1^, and the total pore volume was 0.45 cm^3^ g^−1^. Remarkable micropores and mesopores could be observed in the pore-size distribution curves of MONs-SHs, where micropores occupy a major portion. However, the average pore size was calculated to be about 2.71 nm; hence, the MONs-SH samples prepared in an ethanol–water system were identified as mesoporous materials. FT-IR was employed for the characterization to explore whether -SH is successfully modified in the nanoparticles, as depicted in Figure 1f. The broad absorption peak at 3440 cm^−1^ is attributed to the stretching vibration peak of -OH. The Si-O-Si vibration stretching peak is found at 1000–1100 cm^−1^, and the band located at 910 cm^−1^ belongs to the Si-OH [24,25]. The characteristic absorption peaks of -CH vibration in the organosilicon skeleton are observed in the range of 2900–2980 cm^−1^ and 1410–1420 cm^−1^ [26]. The characteristic absorption peak of MONs-SH at 2580 cm^−1^ is attributed to the -SH stretching vibration, which is not observed in MONs [25]. In addition, the TG analysis results reveal the weight loss of the materials at 30–150 °C, which is attributed to the desorption of physiosorbed water molecules. The obvious weight loss starts at 250 °C, which is ascribed to the decomposition of the MPTMS groups and bridged organic groups. It is worth noting that the weight loss of MONs-SHs at 200–450 °C is significantly higher than that of MONs (Figure 1g). This is mainly attributed to the decomposition of sulfhydryl groups [27,28]. Furthermore, the carbon and sulfur analysis displayed that the S element amount is 10.9 wt% (Table S1). In summary, the successful modification of sulfhydryl groups on mesoporous organosilica nanoparticles could be confirmed.

**Scheme 1:**
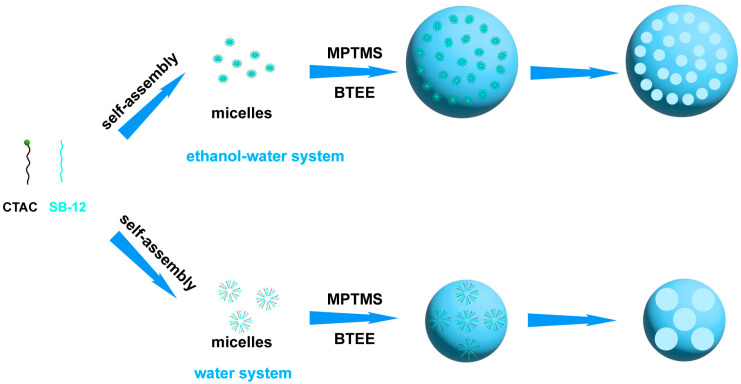
Schematic illustration of preparation of mesoporous organosilica nanospheres in ethanol–water and water systems.

The effect of the reaction system on the preparation of MONs-SHs was investigated during the experiments. Figure S5a exhibits the SEM image of MONs-SHs synthesized in the single water system, showing that the MONs-SHs still maintain spherical morphology. However, the particle size of MONs-SHs is relatively small, and the particle size measured from the TEM image and nano measurer test is only about 18 nm (Figures S5b and S6). The N_2_ adsorption isotherm of MONs-SHs obtained in the single water system delivers the characteristics of the type IV curve with a high adsorption capacity. The average pore diameter is 5.5 nm, and the BET surface area is as high as 749.39 m^2^ g^−1^, as described in Figure S5c. The results of nitrogen adsorption tests suggest that the aqueous system is favorable for obtaining a more pronounced mesoporous structure compared to MONs-SHs obtained in the ethanol–water system. That is, the introduction of ethanol exhibits a pronounced effect on reducing the pore size and enlarging the particle size of the nanoparticles. First, the micelles are formed in a water-ethanol or water system by the self-assembly of the zwitterionic surfactant SB-12 and the cationic surfactant CTAC. Then, mesoporous organosilica nanospheres are formed under the hydrolysis of MPTES and BTEE through the sol–gel process. Interestingly, with the introduction of ethanol into the system, the template (CTAC) could be better dispersed in an ethanol co-solvent, forming fewer micelles. On the other hand, ethanol weakens the hydrophobic and micelle-forming ability of CTAC [29]. Hence, different phase transitions could be induced by ethanol. Ethanol has been confirmed to exhibit a water structure-breaking effect on copolymers, and adding ethanol results in increased copolymer solubility and critical micelle concentration (CMC) [30]. Thus, the CMC in the system is higher than that of the micelles in water, resulting in the rearrangement of surfactants and the formation of nanospheres with larger particle sizes with narrow mesopores [27,31], as depicted in Scheme 1. In addition, the FT-IR analysis of MONs-SHs prepared in the water system is presented in Figure S5d. The absorption peak at 2570 cm^−1^ is attributed to the stretching vibration of -SH, revealing the successful modification of sulfhydryl groups of MONs-SHs prepared in the water system. The MONs-SHs synthesized in the ethanol–water system, which displays a large particle size and ease of separation compared to the synthesized MONs-SHs in the water system, is selected for subsequent adsorption studies towards Pb^2+^ ions.

### 3.2. Adsorption Performances of MONs-SH

The adsorption capacity of the adsorbent is commonly affected by several variables, such as solution concentration, temperature, time, pH, etc. As a result, the above factors were selected in the experiment to investigate the adsorption performance of MONs-SH adsorbent towards Pb^2+^ ions. The concentrations of Pb^2+^ in the solution range from 0 to 0.04 mol L^−1^, and the initial temperature is selected to be room temperature 25 °C.

**Figure 2:**
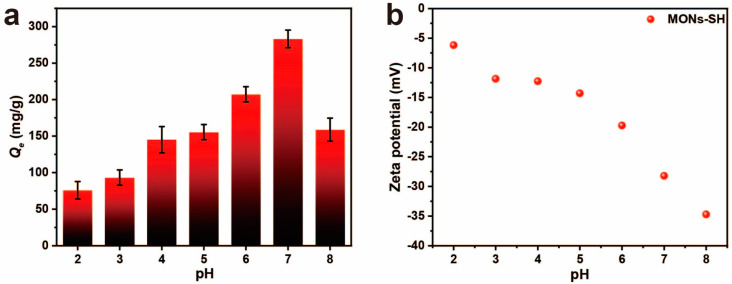
(**a**) Effect of pH value on the adsorption performance of Pb^2+^ on MONs-SHs. Reaction condition: Pb^2+^ solution concentration 0.02 mol L^−1^, adsorbent weight 10 mg, reaction time 60 min, solution volume 10 mL, (**b**) Zeta potential of MONs-SHs under different pH values.

One of the most important variables in determining the adsorption capacity of the adsorbent is the impact of solution pH on the adsorption of Pb^2+^ ions. Hence, during the experiments, we examined the adsorption of Pb^2+^ ions on MONs-SHs by adjusting the pH of the solution. In the experiment, the Pb^2+^ concentration of the solution was 0.02 mol L^−1^, the adsorption time was 60 min, and the pH of the solution varied from two to eight. The experimental results are displayed in Figure 2. With the gradual increase in solution pH (2~7), the adsorption capacity of Pb^2+^ ions on MONs-SHs increases continuously and reaches the maximum value at pH of seven with an adsorption amount as high as 283.17 mg g^−1^. The Zeta potential curves (Figure 2b) under different pH values suggest that the surface charge distribution of MONs-SHs is negative, which is beneficial for the adsorption of metal ions. In addition, the -SH groups uniformly embedded in MONs-SHs belong to soft Lewis bases. In contrast, heavy metal ions such as Pb^2+^ exhibit typical soft Lewis acid properties due to their relatively low electronegativity, high polarizability, and large ionic sizes [32,33]. The super affinity between them endows MONs-SHs with an excellent adsorption capacity for heavy metal ions. Thus, the results reveal that acidic conditions are unfavorable for the adsorption of Pb^2+^ ions on MONs-SHs. The reason for this could be mainly due to the increase in deprotonation and surface electronegativity with the increase in pH value. In addition, high concentration of H^+^ ions under low pH competes with the occupation of reactive adsorption sites of Pb^2+^ ions. The increase in pH from two to seven might lead to an increase in the negatively charged sites of the adsorbent surface, which enhances the attraction between the metal ions and the adsorbent surface, thus improving the adsorption performance of the adsorbent [34]. In contrast, the adsorption of Pb^2+^ ions on MONs-SHs decreases significantly (158.85 mg g^−1^) when the pH of the solution is eight, although the surface charge was negative. This may be attributed to the hydrolysis of the Pb^2+^ ions and the precipitation of lead hydroxides (Figure S7), which prevents the adsorption of Pb^2+^ ions on the adsorbent and leads to a decrease in adsorption performance [35]. Hence, the adsorption of Pb^2+^ ions in alkaline solution would not be applicable as the pH precipitation edge of Pb^2+^ is 7–8.

**Figure 3:**
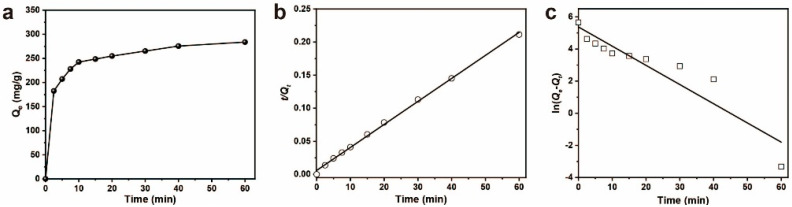
(**a**) Adsorption kinetic curves of Pb^2+^ on MONs-SHs, (**b**) fitted results by the pseudo-second-order kinetic model, (**c**) fitted results by the pseudo-first-order kinetic model. Reaction condition: Pb^2+^ concentration 0.02 mol L^−1^, adsorbent weight 10 mg, reaction time 60 min, solution volume 10 mL.

The influence of reaction time on the adsorption amount and adsorption kinetic process of Pb^2+^ ions on MONs-SHs is further explored in the experiments, and the results are presented in Figure 3. The adsorption capacity of Pb^2+^ ions on MONs-SHs exhibits a rapidly increasing trend within the initial 10 min. Then, the adsorption capacity tends to slow down gradually, and basically reaches equilibrium at about 30 min. Two different kinetic models, the pseudo-first-order (Equation (1)) and pseudo-second-order response models (Equation (2)), are chosen for fitting the data to study the adsorption kinetic process in depth, and the calculated results and parameters are shown in Table 1. Compared with the R^2^ value (0.871) calculated from the primary reaction model, the adsorption kinetics of Pb^2+^ on MONs-SHs is more in line with the secondary reaction model with the R^2^ value of 0.998. In addition, the calculated adsorption amount Q_e_ (285.71 mg g^−1^) is close to the experimental results (283.86 mg g^−1^), further proving that the adsorption of Pb^2+^ on MONs-SHs is a chemisorption process [36].

(1) $$\ln\left(Q_{e}-Q_{t}\right)=lnQ_{e}-k_{1}t$$

(2) $$\frac{t}{Q_{t}}=\frac{1}{k_{2}Q_{e}^{2}}+\frac{t}{Q_{e}}$$

where Q_e_ (mg g^−1^) is the adsorption amount at equilibrium, Q_t_ (mg g^−1^) represents the adsorption amount at any time t, k_1_ (min^−1^) refers to the primary reaction rate constant and k_2_ (g mg^−1^ min^−1^) stands for the secondary reaction rate constant [37].

Different reaction temperatures, including 15, 25, and 35 °C, are selected for the adsorption experiments to investigate the role of temperature in the adsorption of Pb^2+^ ions on MONs-SHs. The thermodynamic parameters, including enthalpy change (ΔH), entropy change (ΔS), and Gibbs free energy change (ΔG) during adsorption are calculated according to the thermodynamic Equations (3)–(5), to evaluate the feasibility of the adsorption process and the direction of the reaction.

(3) $$\Delta G=-RTln\frac{Q_{e}}{c_{e}}$$

(4) ΔG=ΔH−TΔS

(5) $$ln\frac{Q_{e}}{c_{e}}=\frac{\Delta S}{R}-\frac{\Delta H}{RT}$$

where T (K) is the temperature and R (8.314 J mol^−1^ K^−1^) is the molar gas constant [38].

**Figure 4:**
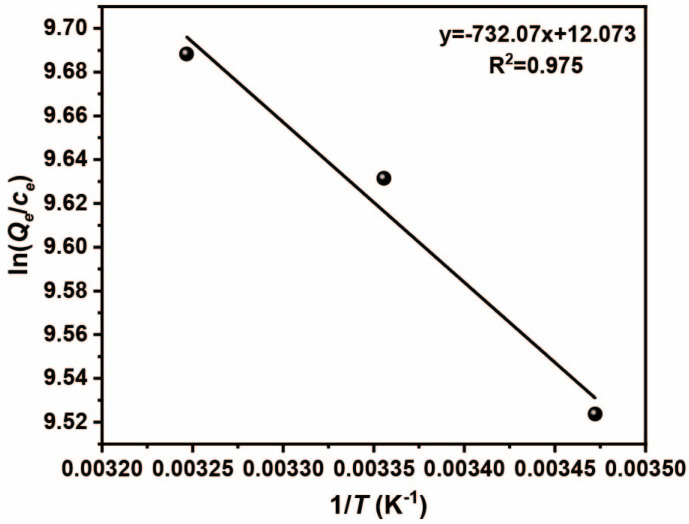
Fitted results by the Van’t Hoff model. Reaction condition: Pb^2+^ concentration 0.02 mol L^−1^, adsorbent weight 10 mg, reaction time 60 min, solution volume 10 mL.

The ΔS and ΔH calculated from the intercept and slope of the straight line based on the Van’t Hoff model by the line fitted according to Equation (5) are described in Figure 4 and Table 2. The positive ΔH value reveals the thermal adsorption process, while the positive value of ΔS confirms that the thermal adsorption process is an entropy-increasing process. As observed, the value of ΔG is less than zero, revealing that the adsorption process is thermodynamically spontaneous. As the temperature increases from 15 to 35 °C, the value of ΔG decreases from −22.82 to −24.82 kJ mol^−1^, indicating that the elevation of temperature favors the adsorption process of Pb^2+^ ions on MONs-SHs.

**Figure 5:**
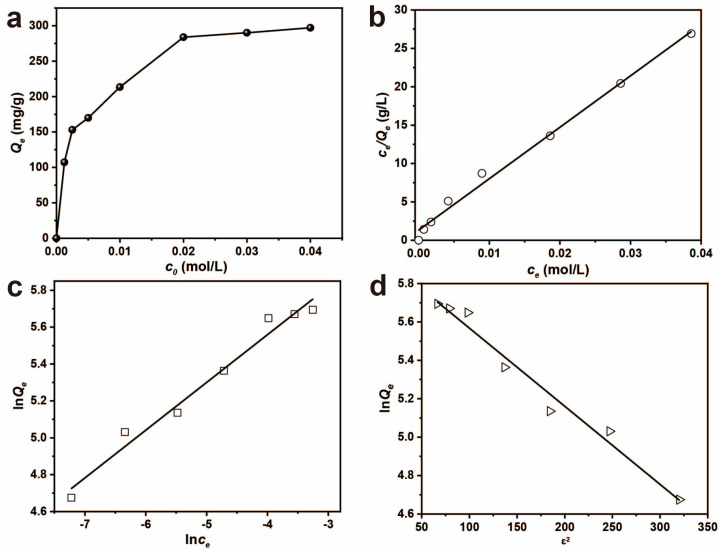
(**a**) Adsorption isotherms of Pb^2+^ on MONs-SHs, (**b**) linear plot of Langmuir model, (**c**) Freundlich model, (**d**) D-R model. Reaction condition: Pb^2+^ solution concentration 0–0.04 mol L^−1^, adsorbent weight 10 mg, reaction time 60 min, solution volume 10 mL.

The adsorption capacity and models of the MONs-SHs towards various concentrations of Pb^2+^ ions are presented in Figure 5. As shown in Figure 5a, the MONs-SHs exhibit a low adsorption capacity at low Pb^2+^ ions concentrations, and the adsorption capacity increases with the increase of Pb^2+^ concentration. When the Pb^2+^ concentration in the solution is 0.04 mol L^−1^, the adsorption amount reaches the maximum value of 297.12 mg g^−1^. To further elaborate the adsorption mechanism of Pb^2+^ ions on MONs-SHs, Langmuir (Equation (6)) and Freundlich (Equation (7)) isotherm models are chosen for the calculations, respectively [32,39].

(6) $$\frac{c_{e}}{Q_{e}}=\frac{1}{Q_{m}K_{L}}+\frac{c_{e}}{Q_{m}}$$

(7) $$lnQ_{e}=lnK_{F}+\frac{{lnc}_{e}}{n}$$

(8) $$lnQ_{e}=lnQ_{m}-K_{D}\varepsilon^{2}$$

(9) $$\varepsilon =RTln(1+\frac{1}{c_{e}})$$

(10) $$E={\left(2K_{D}\right)}^{-0.5}$$

where c_e_ (mol L^−1^) refers to the Pb^2+^ concentration in solution at equilibrium, Q_e_ (mg g^−1^) refers to the amount of Pb^2+^ adsorbed at equilibrium, Q_m_ (mg g^−1^) is the calculated maximum adsorbed amount of the sample, K_L_ (L mmol^−1^) stands for the Langmuir’s constant, n is the non-homogeneity factor in Freundlich’s model, K_F_ represents the adsorption capacity, K_D_ (mol^2^ kJ^−2^) is a constant related to sorption energy, E (kJ mol^−1^) is the free energy of sorption, and ε shows the Polanyi potential.

The correlation coefficient values (R^2^) calculated from different models are deemed important indicators to determine the optimal model. This can accurately describe the adsorption behavior of Pb^2+^ ions on MONs-SHs. The fitting results of Langmuir and Freundlich isotherm models are depicted in Figure 5b,c, respectively, and the corresponding calculated results are summarized in Table 3. The R^2^ fitted from the Langmuir model (0.993) is higher than that (0.977) fitted from the Freundlich model. In addition, the theoretical adsorption amount could be calculated based on the fitting curves, and the value of the Langmuir model (Figure 5b) is 309.11 mg g^−1^, which is closer to the experimental value (Q_(exp)_ of 297.12 mg g^−1^). The result indicates that the adsorption process of Pb^2+^ ions on the sample is inclined to the adsorption on the surface monomolecular layer. In addition, the Freundlich parameter (n) displays the superiority of the adsorption. If n < 1.0, adsorption intensity is favorable over the entire range of concentrations research, and a n > 1.0 suggests that adsorption is beneficial at high concentrations but bad at lower concentrations. Table 3 displays n values > 1.0, indicating that the adsorption intensity is favorable at high concentrations. A higher n means excellent adsorption of MONs-SHs [40,41]. Furthermore, the value of n (n = 3.86) for MONs-SHs in the Freundlich model confirms strong chemical interaction between the MONs-SHs and Pb^2+^.

The D-R model (Dubinin–Radushkevich model) is also used to predict the nature of the adsorption process (physical or chemical adsorption) by calculating the average free energy (E) determined by Equations (8)–(10). E > 16 kJ mol^−1^ means chemical adsorption, 8 kJ mol^−1^ < E < 16 kJ mol^−1^ stands for the adsorption process belongs to the ion exchange mechanism, and E < 8 kJ mol^−1^ represents the physical adsorption [42]. The E value for Pb^2+^ adsorption on MONs-SH is determined to be 11.04 kJ mol^−1^ in Table 3. These results indicate that the Pb^2+^ adsorption process on MONs-SHs mainly occurs through the ion exchange mechanism.

As Langmuir and Freundlich models are not suitable for the pH-dependent adsorption of ions on charged surfaces, equilibrium-based adsorption models, such as surface complexation models (SCMs), have been developed to describe ion adsorption behavior on electrically charged surfaces. The mechanism underlying the adsorption of Pb^2+^ on MON-SHs mainly involves the interfacial equilibrium complexation between the Pb^2+^ species and -SH sites. Hence, a SCM is considered as the correct general solution to simulate and predict the data [43]. However, due to the missing parameters in SCMs at this stage and the complexity of the functional groups, which include -OH and -SH groups on an organosilica surface, we do not adopt a SCM to fit the adsorption curves.

Comparative experiments further demonstrate the importance of the modified sulfhydryl groups for the adsorption of Pb^2+^ ions on MONs (Table S2). The adsorption amount of Pb^2+^ ions on unmodified MONs (96.56 mg g^−1^) is significantly smaller than that of MONs-SHs (297.12 mg g^−1^) in the same Pb^2+^ concentration (0.04 mol L^−1^). This is mainly due to the fact that the modified SH can bind to Pb^2+^ through ion exchange and complexation function [21], which in turn enables MONs-SHs to deliver excellent adsorption performance. The maximum adsorption amounts of Pb^2+^ on several different adsorbent materials are listed in Table 4. The comparison further confirms that MONs-SHs exhibit advantages in the adsorption of Pb^2+^ ions, which is important for the removal of Pb^2+^ from aqueous solutions.

**Figure 6:**
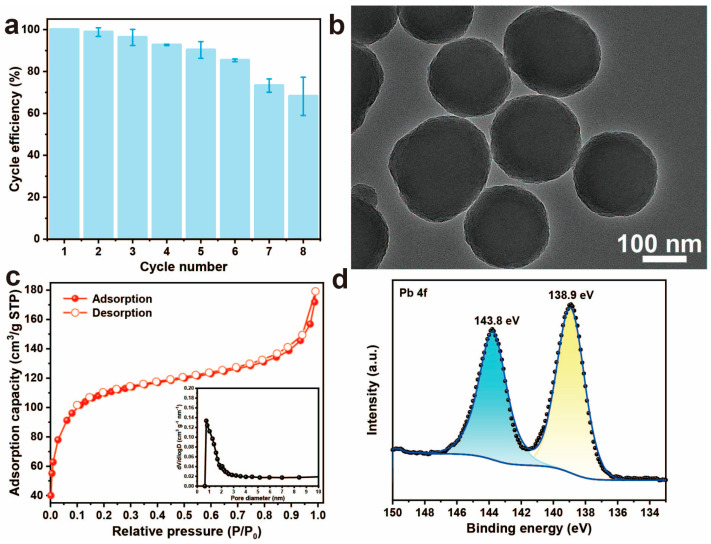
(**a**) Adsorption/desorption experiments to demonstrate the reusability of MONs-SHs for Pb^2+^ ions, (**b**) TEM image, (**c**) N_2_ adsorption isotherm and pore-size distribution curves, and (**d**) XPS spectra of Pb 4f peaks for the MONs-SHs used.

The recycling and re-utilization of the adsorbent is significant in the industrial production. The evolution trend of adsorption efficiency with the cycling number is depicted in Figure 6a. As observed, the adsorption efficiency decreases slightly as the cycling process proceeds. It should be noted that the adsorption efficiency of MONs-SHs is maintained at as high a level as 85.35% in the sixth cycle and decreases to 68.17% in the eighth cycle, revealing a fair recyclability. The reason could be related to the occupation of -SH active sites by the retainment of Pb^2+^ on mesoporous channels, leading to the active sites being blocked during the chemical adsorption of Pb^2+^ ions on MONs-SHs [49]. On the other hand, the partial oxidation of the -SH group during the reusability of the adsorbent would also lower the -SH active sites, as confirmed by Figure S9. Figure 6b describes the TEM image of the used MONs-SHs, which still maintain the original morphology after the eighth cycle. The N_2_ adsorption isotherms of MONs-SHs after eight cycles (Figure 6c) delivers a BET surface area of 413.52 m^2^ g^−1^, an average pore size which is calculated to be about 2.65 nm, and a total pore volume of 0.28 cm^3^ g^−1^, as described in Table S3. The reduction in the BET surface area, average pore size, and total pore volume compared with the original adsorbent is due to the adsorption of Pb^2+^ in the mesoporous channels. XPS analysis is employed to recognize the chemical composition of the MONs-SH after the adsorption of Pb^2+^. The XPS survey of the used MONs-SHs (Figure S8) demonstrates that Pb is obviously present on the surface of MONs-SHs after adsorption. Figure 6d distinctly illustrates the Pb 4f spectrum at 143.8 and 138.9 eV after the adsorption of Pb^2+^, which is indexed to Pb 4f_5/2_ and Pb 4f_7/2_, respectively. The deconvoluted subpeaks with binding energy (BE) interval values of about 4.1 eV between Pb 4f_5/2_ and 4f_7/2_ are assigned to Pb_3_(CO_3_)_2_(OH)_2_ and PbO, respectively. This indicates the coupling of Pb^2+^ on the MONs-SHs, and these two substances are formed during the adsorption process [20,50]. Therefore, it is concluded that the physisorption and chemisorption behaviors perform synergistically in the adsorption of Pb^2+^ ions on MONs-SHs.

## 4. Conclusions

In this study, MONs-SHs are successfully prepared by utilizing CTAC and SB-12 surfactants as dual-template agents via a one-step synthesis strategy. The Pb^2+^ adsorption performance of MONs-SHs is investigated, where the physisorption and chemisorption behaviors perform synergistically in the adsorption of Pb^2+^ ions on MONs-SHs. The introduction of ethanol could significantly increase the particle size of the MONs-SH nanoparticles from 18 to 180 nm without changing the morphology. The modification of sulfhydryl groups favors the adsorption of Pb^2+^ ions on MONs-SHs, where the adsorption capacity reaches as high as 297.12 mg g^−1^ in an aqueous solution. The adsorption of Pb^2+^ on MONs-SHs is more in line with the secondary reaction kinetic model. The corresponding thermodynamic parameter calculations indicate that the adsorption process is spontaneous. This work provides a simple and feasible strategy for the preparation of functionalized mesoporous organosilica nanospheres, which deliver considerable potential applications in the adsorption of Pb^2+^ ions from aqueous solutions.

## Figures and Tables

**Table 1:** Kinetic parameters obtained by the pseudo-second-order and pseudo-first-order rate equations.

Adsorbents	Q_(exp)_ (mg g^−1^)	Model	Parameters		R^2^
MONs-SH	283.86	First-order	Q_e_ (mg g^−1^) 215.21	k_1_ (min^−1^) 0.1195	0.871
		Second-order	Q_e_ (mg g^−1^) 285.71	k_2_ (g mg^−1^ min^−1^) 0.00204	0.998

**Table 2:** Thermodynamic parameters achieved at various temperatures.

ΔH (KJ mol^−1^)	ΔS (J mol^−1^ K^−1^)	ΔG (KJ mol^−1^)		
6.09	100.37	288 K	298 K	308 K
		−22.82	−23.82	−24.82

**Table 3:** Isotherm parameters of Langmuir, Freundlich, D-R, and SCM models.

Values	Models	Parameters		R^2^
Q_(exp)_ (mg g^−1^)	Langmuir	Q_m_ (mg g^−1^) 309.11	K_L_ (L mmol^−1^) 509.36	0.993
297.12	Freundlich	n 3.86	K_F_ 731.50	0.977
	D-R model	Q_m_ (mg g^−1^) 393.39	K_D_ (mol^2^ kJ^−2^) 0.0041	0.977

**Table 4:** Comparison of adsorption capacity of Pb^2+^ on different adsorbents from the literature.

Adsorbents	Concentration	Time (min)	Capacity (mg g^−1^)	Ref.
Mesoporous silica-IP	20 mg L^−1^ Pb^2+^	480 min	52 mg g^−1^	[44]
DSS/MIL-88A-Fe	100 mg L^−1^ Pb^2+^	110 min	250 mg g^−1^	[45]
SBA-15@BDA NPs	100 mg L^−1^ Pb^2+^	60 min	112 mg g^−1^	[46]
Fe_3_O_4_@mSiO_2_-NH_2_	250 mg L^−1^ Pb^2+^	360 min	123.46 mg g^−1^	[47]
FO/MS	100 mg L^−1^ Pb^2+^	60 min	143.47 mg g^−1^	[48]
**MONs-SH**	**0.04 mol L** **^−1^ Pb^2+^**	**60 min**	**297.12 mg g** **^−1^**	**This work**

## Data Availability

Data are contained within the article and Supplementary Materials.

## Supplementary Materials

The following supporting information can be downloaded at: https://www.mdpi.com/article/10.3390/nano15020136/s1, Figure S1. Photographic image of solution (a) before and (b) after titration with EDTA. Figure S2. The particle size distribution of MONs-SH prepared in ethanol-water system. Figure S3. Photographic images of synthetic system adding (a) MPTES, (b) MPTES and BTEE. Figure S4. The EDS spectra of MONs-SH prepared in ethanol-water system. Table S1. The sulfur content of MONs-SH prepared in ethanol-water system. Figure S5. (a) SEM image, (b) TEM image, (c) N_2_ adsorption isotherm, and (d) FTIR spectra of MONs-SH prepared in water system. Figure S6. The particle size distribution of MONs-SH prepared in water system. Figure S7. Photographs of the lead acetate solution in pH = 7 and 8. Table S2. Comparison of adsorption capacity for Pb^2+^ on MONs-SH and MONs. Table S3. The pore information of fresh and used MONs-SH prepared in ethanol-water system. Figure S8. XPS survey of the used MONs-SH. Figure S9. XPS spectra of S 2p peaks for (a) fresh and (b) used MONs-SH.
