# Supplementary material for: Facile Synthesis of Functional Mesoporous Organosilica Nanospheres and Adsorption Properties Towards Pb(II) Ions
Source: Nanomaterials (Basel). 2025 Jan 17;15(2):136. doi: 10.3390/nano15020136 (PMC11767340; doi:10.3390/nano15020136)
Supplement: Supplementary file 1 [file nanomaterials-15-00136-s001.zip › nanomaterials-3295806-supplementary.docx]

**Supporting Information**

Facile Synthesis of Functional Mesoporous Organosilica Nanospheres and Adsorption Properties Towards Pb(II) Ions

Liping Deng ^1,†^, Shichun Gu ^1,†^, Ruyi Wang ^1^, Yapeng He ^2,^*, Hairong Dong ^1^
and Xue Wang ^1,^*

^1^ Yunnan Key Laboratory of Modern Separation Analysis and Substance Transformation, College of Chemistry and Chemical Engineering, Yunnan Normal University, Kunming 650500, China

^2^ Faculty of Metallurgical and Energy Engineering, Kunming University of Science and Technology, Kunming 650093, China

* Correspondence: heyapeng@kmust.edu.cn (Y.H.); xwang@ynnu.edu.cn (X.W.)

† These authors contributed equally to this work.

**Experimental materials**

Cetyltrimethylammonium chloride (CTAC, 97.0%), (3-mercaptopropyl)triethoxysilane (MPTES, 98.0%), sodium acetate (99.0%) and acetic acid (99.5%) were purchased from Macklin Biochemistry Science and Technology Co., Ltd., China. Dodecyl sulfobetaine (SB-12, 98.0%), 1,2-bis(triethoxysilyl)ethane (BTEE, 96.0%) and ethylenediaminetetraacetic acid disodium salt dihydrate (EDTA) were purchased from Shanghai Aladdin Biochemical Technology Co., Ltd., China. Lead acetate (99.5%) was purchased from Tianjin Fengchuan Chemical Reagent Technology Co., Ltd., China. Hexamethylenetetramine (99.0%) and sodium hydroxide (96.0%) were purchased from Xilong Science Co., Ltd., China. Xylenol orange (indicator) was purchased from Lindlarcatalyst, China. Ethanol (99.7%) and concentrated ammonia (25-28%) were purchased from Yunnan Jingrui Science and Technology Co., Ltd., China. Hydrochloric acid (36~38%) and nitric acid (86~97.5%) were obtained from Yunnan Shandian Pharmaceutical Co., Ltd., China. All the above reagents were all analytical reagents without further purification.

**Physical characterizations of materials**

The morphology of the samples was characterized by transmission electron microscopy (TEM) using a JEOL JEM-1400Flash with an operating voltage of 120 kV and scanning electron microscopy (SEM) using a Hitachi Regulus 8100 from Hitachi, Japan. iS20 infrared spectrometer from Thermo Fisher Scientific, USA, was employed to determine the components of the samples within the wavelength range of 400 cm^-1^-4000 cm^-1^. 150-200 mg of the samples were taken and degassed under vacuum at 150 °C for 12 h, and then the samples were subjected to nitrogen adsorption test using the Belsorp Max from Microtracbel, Japan, at 77 K. The samples were analyzed by the Brunauer Emmett Teller (BET) method for specific surface area, Barret Joyner Halenda (BJH) and NLDFT for pore volume and pore size. The thermal stability and composition of the samples at 30-800 °C were tested under N_2_ atmosphere at a heating rate of 10 °C min^-1^ using a synchronous thermal analyzer model TG 209 F1 from Netzsch, Germany. The X-ray photoelectron spectroscopy (XPS) was obtained on a Thermo Fisher ESCALAB 250Xi. The sulfur content was quantified on Leco CS230 carbon and sulfur analyzer apparatus (American).

**Experimental procedures of pH adjusted**

The pH of mixed solution was adjusted to required values (i.e. 2, 3 and 8) with 0.1 M NaOH solution and 0.1 M HCl. Furthermore, the pH= 4, 5 and 6 were adjusted via using buffer system of sodium acetate and acetic acid.

**Reusability of the adsorbent**

0.2 g of MONs-SH was added to the Pb^2+^ solution (0.02 mol L^-1^, 200 mL) at pH 7.0, and the whole reaction time was 60 min. After adsorption Pb^2+^, the MONs-SH was activated by 0.5 mol L^-1^ HNO_3_ for 2 h for reusing in the next cycle. The adsorption-desorption cycles were executed 6 times.


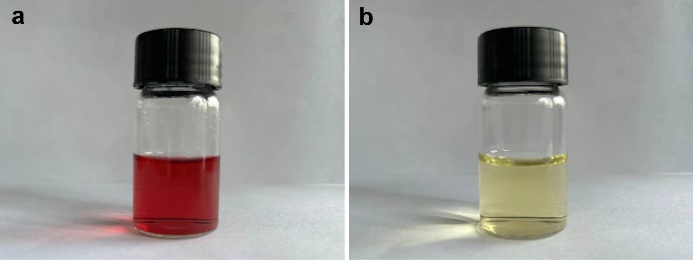


**Figure S1.** Photographic image of solution (a) before and (b) after titration with EDTA


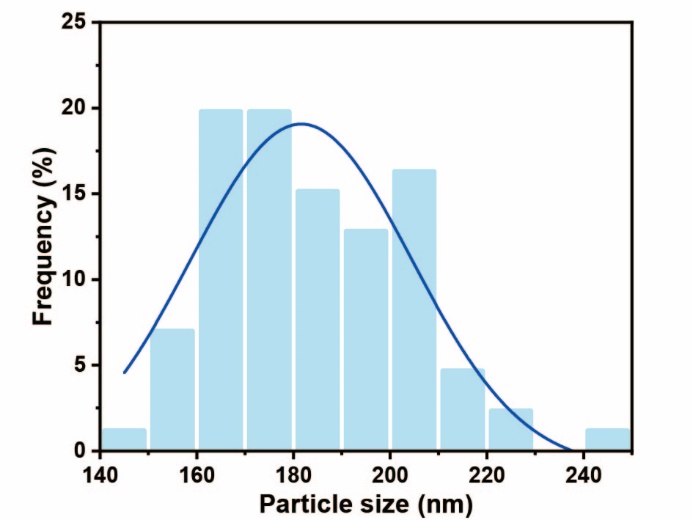


**Figure S2.** The particle size distribution of MONs-SH prepared in ethanol-water system.


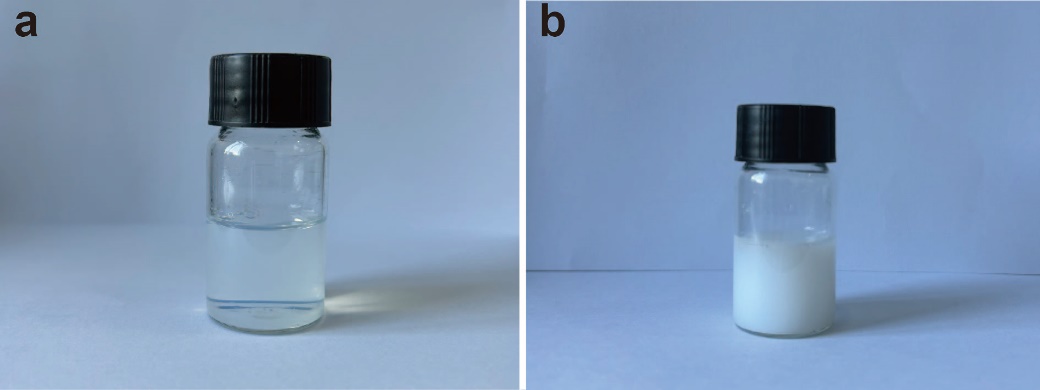


**Figure S3**. Photographic images of synthetic system adding (a) MPTES, (b) MPTES and BTEE.


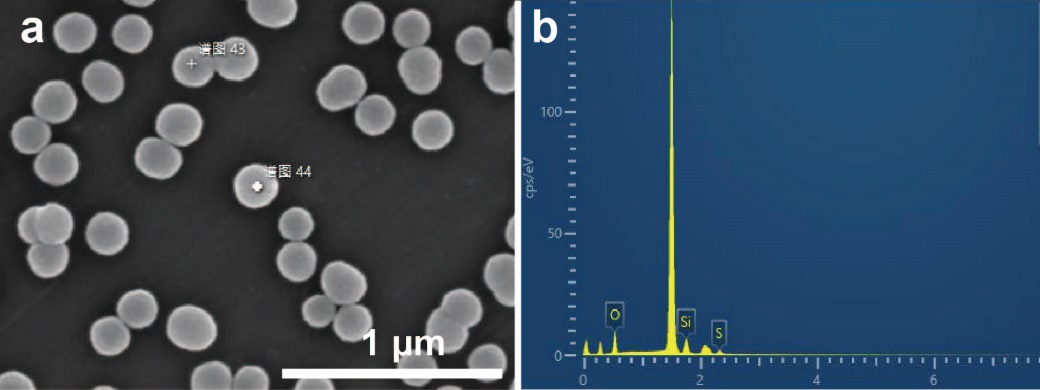


**Figure S4**. The EDS spectra of MONs-SH prepared in ethanol-water system

**Table S1** The sulfur content of MONs-SH prepared in ethanol-water system

| MONs-SH (g) | S (wt%) |
| --- | --- |
| 0.0658 | 10.90% |

**
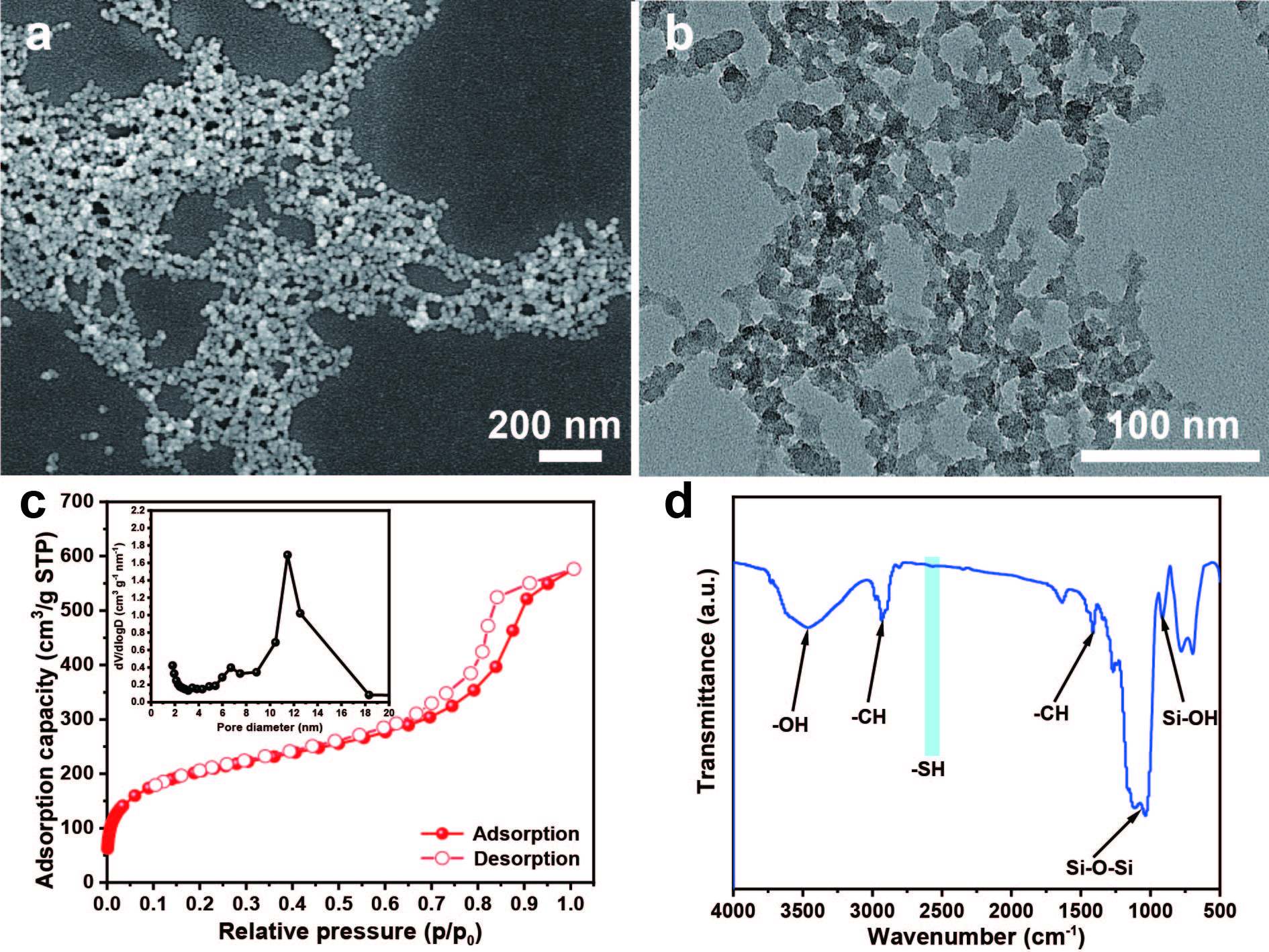
**

**Figure S5.** (a) SEM image, (b) TEM image, (c) N_2_ adsorption isotherm, and (d) FTIR spectra of MONs-SH prepared in water system


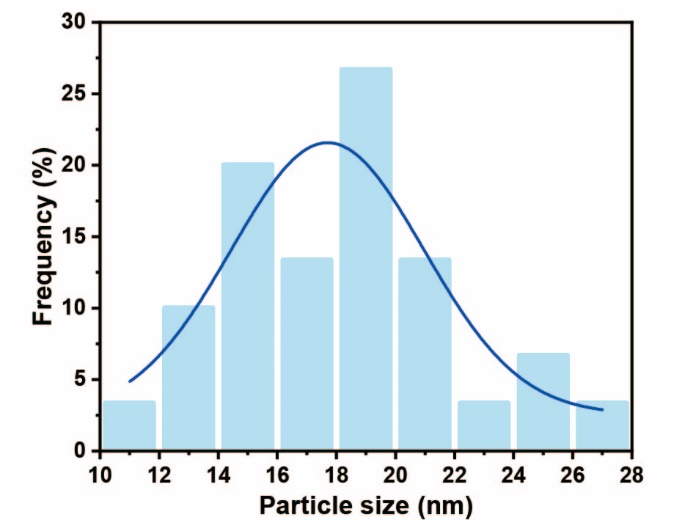


**Figure S6.** The particle size distribution of MONs-SH prepared in water system.


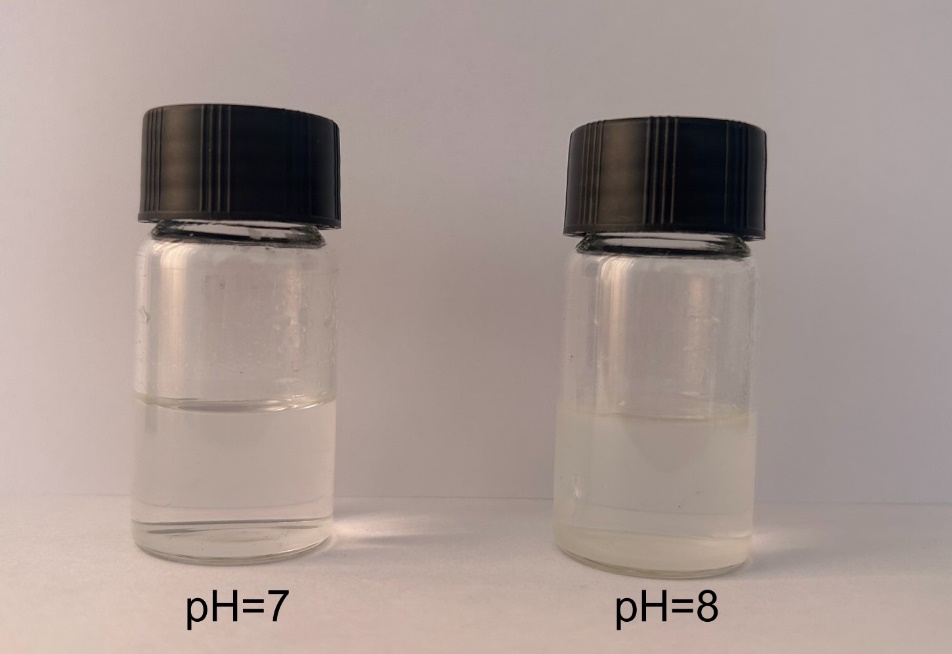


**Figure S7** Photographs of the lead acetate solution in pH= 7 and 8

**Table S2** Comparison of adsorption capacity for Pb^2+^ on MONs-SH and MONs

| Adsorbents | c_0_（mol L^-1^） | Q_(exp)_（mg g^-1^） |
| --- | --- | --- |
| MONs-SH | 0.04 | 297.12 |
| MONs | 0.04 | 96.56 |

**Table S3** The pore information of fresh and used MONs-SH prepared in ethanol-water system

| Adsorbents | BET (m^2^ g^-1^) | average pore size（nm） | total pore volume (cm^3^ g^-1^) |
| --- | --- | --- | --- |
| Fresh | 729.15 | 2.71 | 0.45 |
| Used | 413.52 | 2.65 | 0.28 |


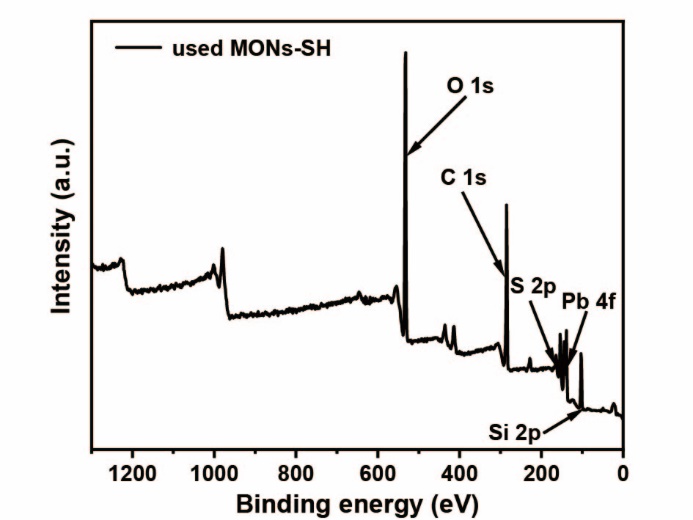


Figure S8. XPS survey of the used MONs-SH


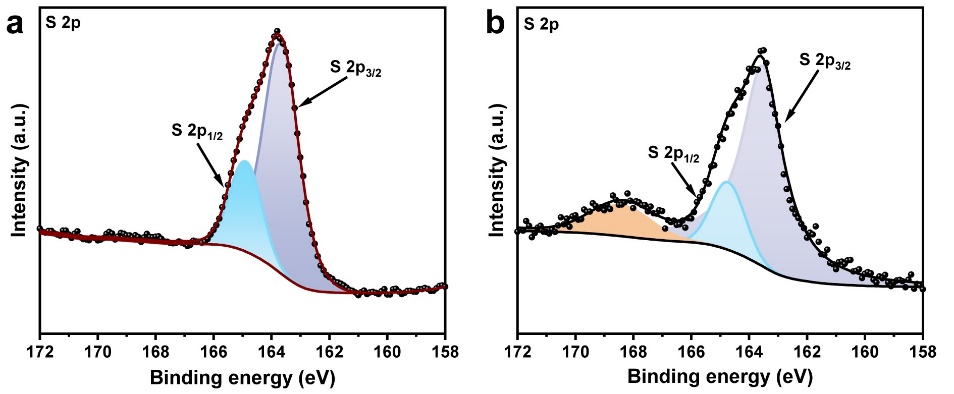


**Figure S9** XPS spectra of S 2p peaks for (a) fresh and (b) used MONs-SH
